# Pulmonary Hyalinizing Granuloma Mimicking Primary Lung Cancer: An Unusual Case Involving a Pulmonary Tumor

**DOI:** 10.1155/2020/3268608

**Published:** 2020-01-21

**Authors:** Hideki Marushima, Hiroki Sakai, Reimi Yoneyama, Hiroyuki Kimura, Tomoyuki Miyazawa, Motohiro Chosokabe, Masahiro Hoshikawa, Koji Kojima, Masayuki Takagi, Hisashi Saji

**Affiliations:** ^1^Department of Chest Surgery, St. Marianna University School of Medicine, Kawasaki 216-8511, Japan; ^2^Department of Thoracic Surgery, Niizashiki Central General Hospital, Saitama 352-0001, Japan; ^3^Department of Pathology, St. Marianna University School of Medicine, Kawasaki 216-8511, Japan

## Abstract

Pulmonary hyalinizing granuloma is a very rare benign condition. This study describes a case involving pulmonary hyalinizing granuloma in a 76-year-old man who presented with a solitary pulmonary nodule, determined through chest radiography and computed tomography, that mimicked primary lung cancer. To establish a definitive diagnosis, tumor resection was performed with histopathological analysis indicating pulmonary hyalinizing granuloma. Radiographic findings in previously reported cases showed that most patients had well-defined margins and usually bilateral, multiple lesions. In our case; however, the solitary ill-defined tumor mimicking lung cancer is an uncommon location for this rare condition.

## 1. Introduction

Pulmonary hyalinizing granuloma (PHG), a rare benign tumorous lesion mimicking a pulmonary neoplasm, occurs as a single or multiple lesions with unilateral or bilateral lung involvement. A definite diagnosis certainly requires pathologic evaluations. PHG is histologically characterized by vitrified collagen fiber bundles and surrounding lymphocytes and plasma cells. Although this disease was first characterized in 1977 by Engleman et al. [1], the details still remain unknown. Nonetheless, PHG has been thought to be associated with mediastinal and retroperitoneal fibrosis as well as autoimmune, hematologic, thromboembolic, and infectious diseases.

## 2. Case Report

**Figure 1:**
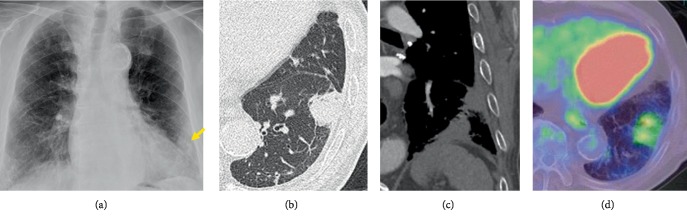
(a) Chest X-ray revealing an irregular shadow in left lower field (arrow). (b) Chest computed tomography (CT) in the lung window revealing a tumor located at S8 of the left lower lobe. (c) Chest CT in the mediastinal window showing a tumor with suspected pleural invasion. (d) Fluoro-2-deoxyglucose-positron emission tomography/CT showing a maximum standardized uptake value of 3.8 in the tumor.

**Figure 2:**
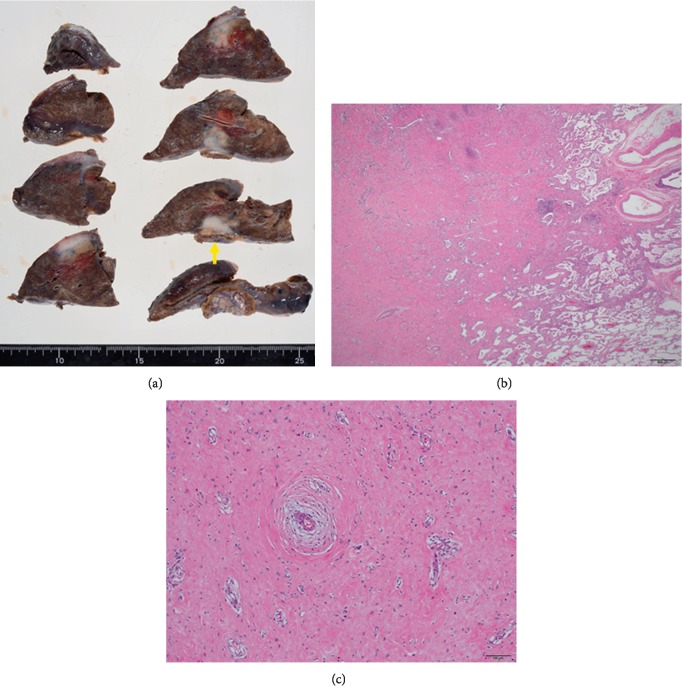
(a) Macroscopic image of the partially resected tumor (48 × 38 × 25 mm) with attaching diaphragm (arrow). (b) and (c) Marked fibrosis, vascular hyperplasia, and necrosis partially accompanied by lymphocytic and histiocytic infiltration.

A 76-year-old man had been previously admitted to another hospital due to subarachnoid hemorrhage and aphasia. His chest X-ray (Figure 1(a)) revealed an irregular solid Tumor shadow along the left lower lung. Chest computed tomography (CT) (Figures 1(b) and 1(c)) revealed a 2.8 cm irregularly shaped tumor with solid component in left S8 area extending to the visceral pleura and diaphragm. Moreover, somewhat diffuse interstitial changes in certain areas were related to interstitial lung disease. CT, fluoro-2-deoxyglucose (FDG)-positron emission tomography (PET)/CT, and brain magnetic resonance imaging findings did not indicate distant metastasis. FDG-PET/CT (Figure 1(d)) revealed increased tumor lesion metabolic activity (maximum standardized uptake value = 3.8). Preoperative CT-guided needle biopsy was then performed at a referral hospital to establish a pathologically definitive diagnosis. After analyzing the preliminary results, adenocarcinoma of the lung was suspected. Therefore, the patient was referred to our university for therapeutic resection of lung carcinoma suspected with a clinical stage of T2a(PL1)N0M0 IB. Following careful medication to prevent acute exacerbation of interstitial pneumonia after surgical treatment, wedge resection of left lower lobe and partial resection were performed. Additional partial resection and reconstruction of the diaphragm were performed due to the tumor attached to it (Figure 2(a)). Pathological findings indicated capillary hyperplasia and remarkable fibrosis (Figures 2(b) and 2(c)) partially accompanied by necrosis and lymphocytic or histiocytic infiltration. Negative results for CK, P40, and TTF-1 immunostaining or the existence of IgG4-positive plasma cells supported the diagnosis of PHG.

## 3. Discussion

PHG is a benign lesion characterized by rich fibrosis. Generally, no specific tendency for age, sex, or race predominance has been observed. Clinical symptoms such as cough, fever, fatigue, dyspnea, chest pain, sinusitis, and pharyngitis are also nonspecifically related to this disease. They may also be related to primary diseases such as mediastinal and retroperitoneal fibrosis, autoimmune diseases, tumors, or infectious diseases that have been associated with PHG [2].

After searching the MEDLINE database of the National Library of Medicine was searched for relevant literature using the key words “hyalinizing granuloma” and “Japan,” 20 patients from Japanese literature were reviewed (Table 1). Accordingly, the mean age at PHG diagnosis was 46 years (range: 28–65 years) with male predominance being observed (12 males/8 females). Among the patients, 14 (70%) were asymptomatic, three (15%) reported respiratory symptoms and anemia, and five (25%) exhibited comorbidity, which comprised initial lung squamous carcinoma, interstitial pneumonia, collagen disease suspicious, and hypergammaglobulinemia.

Preoperative biopsy was performed in 14 patients (70%), none of whom were diagnosed with PHG. Surgical resection was conducted in 12 patients who had suspected lung cancer or thymoma, diagnosed lung cancer or metastatic lung tumor, and undiagnosed diseases. As suggested in other studies, diagnosing PHG through preoperative biopsy is difficult [3–5]. In the present case, the dysplastic area of alveolar epithelial cells in the alveolar epithelium between the hyalinized area and the normal area was biopsied under CT guidance. Considering that the biopsy sample revealed no tumor site and only alveolar epithelium dysplasia, a diagnosis of adenocarcinoma was suspected. Nonetheless, reports have recommended surgical biopsy given possibility that only the inflammation site may be included when attempting tumor site biopsy.

In conclusion, PHG can be misdiagnosed given its complex tissue components. To establish the appropriate diagnosis, surgery while considering differential diagnoses is essential.

## Figures and Tables

**Table 1:** Summary of the 20 pulmonary hyalinizing granuloma cases reported in Japan.

Variables		Number
Mean age (range)	46	(28–65)
Gender	Male	12 (60%)
	Female	8 (40%)
Single or multiple	Single	4 (20%)
	Multiple	16 (80%)
Comorbidity	Primary lung squamous cell carcinoma	1 (5%)
	Emphysematous merger interstitial pneumonia	1 (5%)
	Collagen disease	1 (5%)
	Hypergammaglobulinemia	1 (5%)
	Non	15 (75%)
Preoperative diagnosis	Lung cancer	1 (5%)
	Lung cancer suspicion	1 (5%)
	Metastatic lung cancer suspicion	2 (10%)
	Thymoma suspicion	1 (5%)
	None	15 (75%)
Surgical procedure	Lobectomy	5 (25%)
	Partial excision	7 (35%)
	Follow up	5 (25%)
Radiological finding	FDG-PET/CT performed in 4 cases including this case	
	SUV max range 2.3–9.5	

## References

[1] Engleman P., Liebow A. A., Gmelich J., Friedman P. J. (1977). Pulmonary hyalinizing granuloma. *The American Review of Respiratory Disease*.

[2] Na K. J., Song S. Y., Kim J. H., Kim Y. C. (2007). Subpleural pulmonary hyalinizing granuloma presenting as a solitary pulmonary nodule. *Journal of Thoracic Oncology*.

[3] Brandao V., Marchiori E., Zanetti G. (2010). Hyalinizing granuloma: an unusual case of a pulmonary mass. *Case Reports in Medicine*.

[4] Duzgun N., Kurtipek E., Esme H., Eren Karanis M. I., Tolu I. (2015). Pulmonary hyalinizing granuloma mimicking metastatic lung cancer. *Case Reports in Pulmonology*.

[5] Lhote R., Haroche J., Duron L. (2017). Pulmonary hyalinizing granuloma: a multicenter study of 5 new cases and review of the 135 cases of the literature. *Immunologic Research*.

